# Impact of the demographic and aetiological factors and intraoperative findings on postoperative outcomes in chronic otitis media surgery

**DOI:** 10.3906/sag-1907-125

**Published:** 2020-02-13

**Authors:** Süleyman CEBECİ, Mehmet Suat ÖZBİLEN, İsmet BAYRAMOĞLU, Yusuf Kemal KEMALOĞLU, Kadir Kemal UYGUR, Yıldırım Ahmet BAYAZIT, Recep KARAMERT

**Affiliations:** 1 Department of ENT, Faculty of Medicine, Gazi University, Ankara Turkey; 2 Department of ENT, Eryaman Private Hospital, Ankara Turkey; 3 Department of ENT, Faculty of Medicine, Medipol University, İstanbul Turkey

**Keywords:** Chronic otitis media, cholesteatoma, tympanoplasty, surgery

## Abstract

**Background/aim:**

Surgical success is related with many factors belonging to both the patient and the disease. This study aims to analyse the preoperative and intraoperative characteristics, the postoperative results, and the factors affecting the surgical success in different types of chronic otitis media (COM).

**Materials and methods:**

A total of 1510 ears of 1398 patients who underwent COM surgery were included in the study. Postoperative results were obtained from 376 ears of 356 patients who had been followed after surgery. The demographic characteristics of the patients, such as age and sex, operative findings, preoperative audiological examination results, and final audiometric and otoscopic examination findings, were retrospectively obtained from the archives of the department.

**Results:**

The most frequent diagnosis was simple COM (39.9%), and the most frequently performed surgery was tympanoplasty without mastoidectomy (46.6%). The overall hearing success rate was found to be 75.8%. Postoperative hearing success was significantly associated with the chronic otitis subgroup, ossicular pathologies, and the condition of the middle ear mucosa. Postoperative graft take rate was found to be 78.6%. Graft success was statistically significantly higher in patients with normal middle ear mucosa. Performing mastoidectomy, the presence of patency in aditus ad antrum, and being a paediatric case had no impact on graft success.

**Conclusion:**

Factors affecting the success of COM surgery include age, chronic otitis subgroup, location and size of perforation, the condition of the middle ear mucosa, and the level of the ossicular disease. These factors should be known and an appropriate treatment plan should be prepared.

## 1. Introduction

Chronic otitis media (COM) is the chronic inflammation and the infection of the middle ear and mastoid space characterized by perforation of the tympanic membranes and purulent discharge from the external ear canal [1]. COM is characterized by perforation of the tympanic membranes, intermittent ear discharge, and hearing losses, which usually lasts more than 3 months and does not recover completely with medical treatment. The aim of chronic otitis surgery is the clearance of the disease in the middle ear and mastoid bone, achievement of tympanic ventilation, reconstruction of the sound conduction mechanisms, and the formation of a dry and self-cleaning cavity [2].

Achievement of the expected success in surgical treatment is not only related to the surgeon; it also depends on many factors related to the patient. Factors such as age, sex, previous surgery, status of the middle ear mucosa, ossicles, presence of ear discharge, cholesteatoma, tympanic membrane perforations, and tympanosclerosis were examined in several previous studies for different COM types [3–6]. The impacts of these factors on functional and morphologic outcomes in COM surgery were evaluated in the literature [7–10]. Differently in this study, COM surgery was evaluated for all aspects such as preoperative features of the patients, operative findings, postoperative outcomes, and related factors in a large case series. All COM types were also classified and investigated in detail. 

This study aims to analyse the effects of the factors related to the disease, surgery, and the patient on the surgical success in different types of COM and share our clinical experiences in COM surgery.

## 2. Materials and methods

In this study, 1510 ears of 1398 patients who underwent COM surgery between January 2007 and June 2012 in the Department of Otorhinolaryngology of the Faculty of Medicine at Gazi University were analysed regarding their preoperative and intraoperative characteristics. The postoperative results of 356 patients with follow-up findings were included in the study. Before the study, ethical approval was obtained from the Local Ethics Committee of the Faculty of Medicine of Gazi University (285/27.06.2012).

Demographic characteristics such as age and sex, operative findings, preoperative audiological examination results, and final audiometric and otoscopic examination findings of the patients were obtained from the archives and the electronic database of the department.

The patients were divided into 6 subgroups according to the operative findings. The patients with a perforated tympanic membrane having a healthy middle ear mucosa without discharge were evaluated as having simple COM. The patients with perforated tympanic membrane and oedematous, hypertrophic, and wet middle ear mucosa were evaluated as having chronic mucosal otitis. The patients with cholesteatoma were considered to have chronic otitis with cholesteatoma, and cholesteatoma was divided into 3 subgroups of attic retraction cholesteatoma, sinus tympani retraction cholesteatoma, and pars tensa total cholesteatoma dependent on anatomical spread [11]. The cases of tympanosclerosis and tympanosclerosis sequel were accepted as chronic otitis with tympanosclerosis. Patients with tympanic retraction pockets and adhesion were evaluated as having chronic otitis with retraction and adhesive otitis.

The type of the operation performed, the pathologies and anomalies (e.g., Körner’s septum, sclerotic mastoid, anteriorly displaced sigmoid sinus) encountered during the operation, the condition of the eardrum and ossicular chain, whether ossiculoplasty was performed, and the type of graft material used were obtained from the patient charts. The anatomic and pathologic operative findings were compared in different chronic otitis types. 

Patients were divided into 3 groups according to their ages. Children up to the age of 16 were considered as the children’s group; patients aged between 16 and 65 years and those over 65 years of age were considered as the adult and old-age groups, respectively.

The preoperative and postoperative hearing levels of the accessible patients were recorded by calculating the pure tone averages and the air-bone gaps at 500 Hz, 1000 Hz, and 2000 Hz in the audiometric examinations. 

In accessible patients, postoperative outcomes and the related factors were analysed. Patients without any pathological findings such as perforation, adhesion, lateralization in their grafts, and disease recurrences were accepted as having successful morphologic outcomes. The most recently performed audiometry (at least 2 months following the surgery) was evaluated, and 10 dB HL hearing gain or air-bone gap of 20 dB HL or less in postoperative audiometry was accepted as successful in hearing outcomes [12].

The postoperative outcomes and the factors affecting the hearing success such as aetiology, preoperative hearing level, condition of middle ear mucosa, and ossicle pathologies were compared. Graft take rate was calculated and the effects of age, middle ear mucosa, performing mastoidectomy, patency of aditus ad antrum, and site of perforation on graft success analysed. In addition, MERI (Table 1) [13] scores were calculated for accessible patients as numeric indicators of the severity of middle ear disease and we analysed the impact on anatomic and functional results.

**Table 1:** Middle ear risk index.

Risk factor	Risk value
Otorrhoea I Dry II Occasionally wet III Persistently wet IV Wet, cleft palate	0123
Perforation Absent Present	01
Cholesteatoma Absent Present	01
Ossicular status O: M + I + S+ A: M + S+ B: M + S- C: M – S+ D: M – S– E: Ossicle head fixation F: Stapes fixation	0123423
Middle ear granulation or effusion No Yes	01
Previous surgery None Staged Revision	012
MERI 0, normal; MERI 1–3, mild diseases; MERI 4–6, moderate diseases; MERI 7–12, severe diseases.	

The data were analysed using SPSS 16.0 (SPSS Inc., Chicago, IL, USA). A normal distribution of the quantitative data was checked using the Kolmogorov–Smirnov test. The parametric tests were applied to data of normal distribution, and nonparametric tests were applied to data of questionably normal distribution. Pearson’s r was used to calculate the correlation coefficients. The distribution of categorical variables in both groups was compared using the Pearson chi-square test. 

## 3. Results

The mean age of the patients was 33.81 (SD = 14.715) years, and the median age was 32 (range: 5–75) years. Furthermore, 46.1% of the patients were male and 53.9% were female, and 50.3% of the left and 49.7% of the right ears were operated on. Primary (n = 1229: 85.6%) and revision (n = 281: 14.4%) surgeries were performed.

Considering the preoperative and intraoperative findings, the majority of the cases were diagnosed as simple chronic otitis, as shown in the Figure. In otomicroscopic examination, central perforations (n = 681: 45.5%) were most frequently encountered (Table 2).

**Figure 1:**
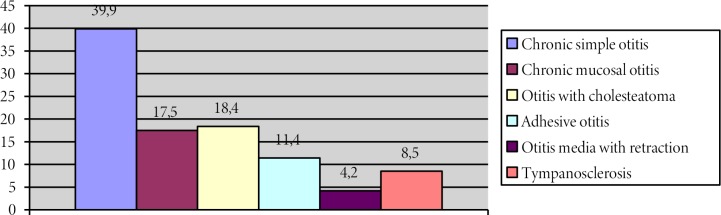
The distribution of COM subgroups.

**Table 2:** The condition of the eardrum in the preoperative examination.

Condition of the eardrum	n	%
Attic perforation	12	0.7
Central perforation	681	45.5
Anterior quadrant perforation	80	5.3
Posterior quadrant perforation	128	8.4
Subtotal perforation	215	14.2
Total perforation	23	1.5
Retraction/adhesion	338	22.3
Other (polyp, intact eardrum)	33	2.1
Total	1510	100

The most frequently performed surgery was tympanoplasty without mastoidectomy (n = 674: 46.6%). The open cavity surgeries (n = 274: 18.2%) were preferred especially in cholesteatoma patients. 

Complete or incomplete Körner’s septum was detected in 23.7% (n = 198) of the patients who underwent mastoidectomy. Körner’s septum was seen significantly more frequently in patients with adhesive otitis and cholesteatoma relative to other chronic otitis subgroups (P = 0.0001). Sclerotic mastoid (25%) and anteriorly displaced sigmoid sinus (14%) were also seen at a significantly higher rate in the adhesive otitis and cholesteatoma patient group than the other chronic otitis subgroups (P = 0.0001).**** The incidence of lateral semicircular canal (LSSC) dehiscence was found to be 3.1% (n = 26) in all mastoidectomized patients and was significantly higher in patients operated on for cholesteatoma (8.6%) compared to the other chronic otitis subgroups (P < 0.05).

In 10.2% (n = 85) of the patients who underwent mastoidectomy, an eroded facial canal was seen due to the disease, and in 1.1% of cases, facial canal dehiscence was detected. In 2 cases, the facial nerve was damaged iatrogenically. An eroded facial canal was observed in 28.5% of cholesteatoma cases (P = 0.0001). 

Ossicular chain pathologies were detected in 687 patients (45.5%) and the remaining 823 (54.5%) patients had intact ossicular chains. These pathologies were seen in the incus (94%), stapes (47.5%), and malleus (33.4%), respectively, in the patients who had ossicular chain pathologies. Based on eardrum findings in our otoscopic examinations, the ossicular pathologies were most commonly associated with total perforations (P < 0.05). Considering the aetiologies, the ossicular chain pathologies were most frequently observed in chronic otitis cases with cholesteatoma. When the ossicular pathologies were compared according to the cholesteatoma types, stapes injury was most frequently seen in cases of sinus tympani cholesteatoma (47.3%) (P = 0.0001).

The cases of chronic otitis with cholesteatoma were classified as attic retraction (n = 114: 41%), sinus tympani retraction (n = 52: 18.7%), and pars tensa (n = 580: 20.8%) cholesteatoma. The remaining cases of cholesteatoma consisted of recurrent, residual, and iatrogenic cholesteatomas encountered in the revision surgeries.

In 129 (8.5%) cases, surgery had been performed in chronic otitis with tympanosclerosis patients and 69.8% of these patients were female. Stapes fixation was detected in 63.6% of the cases of tympanosclerosis. 

In all cases except radical mastoidectomy, tympanoplasty was performed for the repair of perforation and reinforcement. The classification of tympanoplasties was essentially based on the Tos classification [11]. Type 1 (n = 1016: 69.4%), 2 (n = 302: 20.6%), 3 (n = 120: 8.2%), 4 (n = 23: 1.6%), and 5 (n = 3: 0,2%) tympanoplasties were performed for the indicated percentages of patients. An over-underlay technique was used in 86.1% of cases. The most commonly used graft material was temporal muscle fascia, which was used in 90.4% of cases (Table 3).

**Table 3:** The distribution of the graft materials used.

Graft material	n	%
Formaldehyde treated temporal fascia	435	28.8
Nonformaldehyde treated temporal fascia	881	58.4
Periosteum	8	0.5
Cartilage + perichondrium	118	7.9
Tutoplast graft	15	0.9
Other	7	0.4
Graft unused	46	3.1
Total	1510	100

Ossicular chain reconstruction was performed in 33.6% (n = 507) of cases. The most commonly used technique was the interposition of the incus.

Analysis of the postoperative results was performed for 376 ears of 356 patients who had accessible follow-up information. The follow-up period ranged from 2 to 48 months and the median follow-up period was 10 months. The majority of patients (n = 125: 33.2%) were diagnosed with simple chronic otitis. The most common operation was tympanoplasty without mastoidectomy (n = 145: 38.8%) and 132 (35.2%) patients underwent ossicular chain reconstruction.

In this group, hearing success rate was found to be 75.8%. The postoperative hearing success was significantly associated with the chronic otitis subgroup (P < 0.05). Accordingly, the hearing gain was at a maximum in patients operated on for simple COM, while it was lower in cases of adhesive otitis and cholesteatoma. The postoperative hearing success was lower in cases of sinus tympani cholesteatomas (38.8%) compared with attic (70%) and pars tensa (61.5%) cholesteatomas (P = 0.0001).

In cases in which the middle ear mucosa was healthy, the postoperative hearing results were found to be more successful compared to cases involving oedema and hypertrophic mucosa (P = 0.027). The postoperative hearing results were found to be more successful in cases in which stapes were intact (P = 0.0001).

Ossiculoplasty was performed for the reconstruction of conduction in 132 of 376 cases. A total of 85 (64.4%) patients underwent reconstruction with autologous ossicles, and 56 (65.8%) of these procedures were successful. In 10 (0.7%) cases, artificial prostheses (total ossicular replacement prosthesis (TORP), partial ossicular replacement prosthesis (PORP)) were used to improve the hearing and 8 (80%) of these procedures were successful. A total of 33 patients underwent reconstruction with cortical bone and 28 (84.8%) of these procedures were successful. There was no significant difference between the materials used for ossicular reconstruction regarding postoperative hearing success (P = 0.226).

In 320 (85.1%) of 376 cases, a closed technique was preferred, while 56 (14.9%) patients underwent an open technique surgery. The postoperative hearing success rate was significantly higher with the closed technique (80%) compared to the open technique (51.8%) (P = 0.0001).

No recurrence was seen in 78.6% of cases and postoperative graft success was achieved in these patients. However, 4.5% of the patients developed adhesions, and in 16.6% of them, tympanic perforations reoccurred. Rates of graft success and disease control in central perforations were found to be higher than in cases of the other types of perforation (P = 0.014). In patients with normal middle ear mucosa, graft success was statistically significantly higher than those with wet-oedematous and hypertrophic mucosa (P = 0.0001). Performing mastoidectomy or the presence of patency in aditus ad antrum had no impact on graft success (P = 0.707 and P = 0.112, respectively). It was seen that graft success was significantly lower in patients over 65 years of age compared to younger patients (P = 0.038), and similar results were obtained for the paediatric cases and the younger adult patient group.

According to the MERI scores, 51.3% (n = 193) of accessible patients had mild (MERI 1–3) disease, 36.4% (n = 137) had moderate (MERI 3–6) disease, and 12.2% (n = 46) had severe disease, respectively. It was seen that graft success and hearing success significantly decreased as MERI scores increased (P = 0.013 and P = 0.0001, respectively) (Table 4). For the patients with mild disease (MERI 1–3) the most commonly performed operation was tympanoplasty without mastoidectomy, for the patients with moderate disease (MERI 3–6) the most commonly performed operation was mastoidectomy + tympanoplasty, and for severe disease (MERI 7–12) the most commonly performed operation was modified radical mastoidectomy (P = 0.0001).

**Table 4:** The comparison of anatomic and functional results considering the MERI scores.

MERI	Anatomicsuccessa (%)	Functional successb (%)
Mild (MERI 1–3)	83	90.2
Moderate (MERI 3–6)	77.7	64.2
Severe (MERI 7–12)	68.3	50

a: x2 = 16.071, P = 0.013, b: x2 = 48.36, P=0.000.

## 4. Discussion

This study gives comprehensive information about COM in a large case series. Intraoperatively encountered anomalies and pathologic findings are important in COM surgeries. While some findings are related to the aetiology of the diseases, others occur as a result of the diseases.

Körner’s septum is a bony laminate separating the antrum and mastoid cells [14]. According to Proctor, Körner’s septum causes the blockage of the attic and aditus and so predisposes to infection in mastoid cells [15]. Göksu et al. encountered Körner’s septum in 20.93% of 688 mastoidectomy operations and in 54.68% of these patients the aetiologic diagnosis was reported to be caused by ventilation problems such as retraction pouch and adhesive otitis [16]. In our case series, similarly, the incidence of Körner’s septum was found to be 24.7%, and these patients were most commonly operated on for adhesive otitis and then for cholesteatoma. 

The incidence of intraoperative facial canal dehiscence in the literature varies according to the aetiology, being reported in a range between 0.5% and 33% [17–20]. In our cases, spontaneous dehiscence in the facial canal was detected in 1.1% of cases and disease-related facial canal erosion in 10.2% of cases, which increased up to 28.5% in cases of cholesteatoma. According to our study and the literature, facial canal dehiscence is not less frequent, so it is always necessary to work carefully during middle ear and mastoid surgery. 

Labyrinthine fistula is most frequently encountered in cholesteatoma and can be seen in 1 of 10 cases. The treatment of the fistula should be left for the end of the operation, regardless of whether it is an open or closed technique, and the perilymph should not be aspirated [21]. In our study, LSCC dehiscence was observed in 3.1% of the patients who underwent mastoidectomy and was repaired by closure with temporal fascia and bone.

When the literature is reviewed, the rates of 35%–42.5% have been reported for ossicular chain defects in cases of COM [22,23]. In order of their decreasing rates of incidence, ossicular chain defects are listed as necrosis of the long process of the incus, the loss of incus and suprastructure of stapes, and loss of all ossicles except stapes footplate [24]. In our study, pathologies in the incus (42.8%), stapes (21.7%), and malleus (15.2%) were detected in the indicated percentages of cases. In our series, ossicular chain pathologies were most common in cases of chronic otitis with cholesteatoma.

Vartiainen and Nuutinen categorized the types of cholesteatoma according to the Tos classification [11] and detected attic retraction cholesteatoma in 44%, sinus tympani retraction cholesteatoma in 34.8%, and pars tensa cholesteatoma in 21.1% of 431 ears [3]. On the basis of the Tos classification, the most common type of cholesteatoma was attic retraction cholesteatoma (41%) in our cases.

Debates on the use of open or closed techniques in the treatment of cholesteatoma are still ongoing today. The closed techniques are becoming more popular due to the easier postoperative care, no need for protection from water contact, and better auditory outcomes [5]. However, many surgeons reported high residual and recurrence rates in closed techniques and stated that these cases require a second-look operation [25,26]. Vartiainen and Nuutien have suggested an open technique in cholesteatomas reaching the attic and extending behind the facial canal and aditus [3]. They suggested a closed technique in cases of limited sinus tympani and pars tensa cholesteatomas and limited cholesteatoma without any pathology in the mastoid [3]. There is no routine practice in cholesteatoma surgery. The technique to be selected depends on the patient, the disease, and the experience of the surgeon. In our clinic, an open technique is frequently preferred in cholesteatoma surgery. In this study most (68.7%) of the cholesteatoma patients underwent open technique tympanomastoidectomy. 

Tympanosclerosis is a disease characterized by the storage of progressive hyaline material due to the chronic inflammation of the eardrum and middle ear, and hypertrophy of the middle ear submucosa [4]. The calcifications around the eardrum and the ossicles lead to fixation, resulting in conductive and mixed type hearing loss. In the literature, the rate of tympanosclerosis in patients operated on for chronic otitis has been reported to range between 5.5% and 11.6%, and it is more frequently diagnosed in female patients [27,28]. In our study, the rate of tympanosclerosis was found to be similar with the literature (8.5%) with a female predominance (69.8%).

In patients with mucosal disease, as the severity of the disease increases, the hearing results are reported to be worse than in those with normal mucosa [29,30]. In our study, hearing success rates in patients with healthy middle ear mucosa were found to be higher than in patients with oedematous and hypertrophic mucosa (P = 0.027).

The state of the stapes is an important factor affecting postoperative hearing gain. In cases lacking the stapes suprastructure, the hearing results are worse than in cases of intact stapes [31]. According to our study, the presence of the stapes significantly affects postoperative hearing results (P = 0.0001).

The hearing results are considered to be better with closed techniques [32]. Vartiainen and Nuutinen reported that in cases of open cavity tympanomastoidectomy, hearing improved in 1/3 of the cases, did not change in 1/3 of the cases, and worsened in 1/3 of the cases [3]. As we have seen, it is obvious that hearing success is better with closed techniques, but the condition of the middle ear mucosa and stapes affects the hearing more severely than the presence or absence of the canal.

Patients with chronic otitis with cholesteatoma are reported to have worse hearing results [25]. In our case series, the hearing success rate in patients with cholesteatoma was found to be low (61.5%). The least successful hearing results were found for sinus tympani cholesteatomas (38.5%). 

The technique to be preferred for the reconstruction of hearing is closely related to the state of the ossicles. The most frequently encountered pathology is the erosion of the long process of the incus [29]. In this case, incus interposition, or in cases in which the incus is affected intensively by the disease, cortical bone interposition can be applied [7]. Austin reported that he had better results using cortical bone rather than eroded ossicle [33]. In our study, hearing success was achieved in 28 (84.8%) of the 33 patients who underwent reconstruction with cortical bone, while hearing success was achieved in 56 (65.8%) of 85 patients who underwent reconstruction with incus. The use of cortical bone was found to be substantially successful.

The second most frequent pathology in ossicular chain defects is the erosion in the long process of the incus and the crura of the stapes. In this case, either the incus of the patient or an artificial prosthesis may be used. Slater et al. reported success rates of 81% and 67% in 250 patients using PORP and TORP, respectively [34]. In our study, artificial prostheses (TORP, PORP) were used in 10 of 130 patients (0.7%) who underwent hearing reconstruction. We were successful in 8 (80%) of these cases that involved hearing reconstruction using a prosthesis. 

Various graft materials such as cartilage, perichondrium, loose connective tissue, fat, veins, homologous dura mater, and temporal muscle fascia are used for tympanoplasty [35]. The success rates of tympanoplasties using temporal fascia in the literature vary between 24% and 90% [35]. The factors affecting the success rate are listed as age, location and size of the perforation, the status of the ear before the operation, Eustachian tube function, the status of the opposite ear, and the graft material used [36]. The success rate in our series was found to be 78.6% in 369 cases. Graft success was higher in patients with chronic otitis with cholesteatoma, retraction, and tympanosclerosis, but lower in cases of mucosal otitis and adhesive otitis.

The general opinion is that anterior and total perforations negatively affect graft success compared to central perforations. In the literature, there are studies indicating that the nutritional supply of the anterior quadrant of the eardrum is weaker and makes the achievement of graft take success more difficult in this quadrant [37]. In our study, as in the literature, it was found that the graft take success rates in central perforations were significantly higher relative to the anterior quadrant and total perforations.

One of the factors affecting the success of tympanoplasty is the condition of the middle ear mucosa. Many surgeons expect the middle ear to dry before the operation, but this is not always possible. According to Paparella et al., repair of the eardrum should be done in dry ears [38]. In our study, those with normal middle ear mucosa were found to have significantly higher graft success rates compared to those with wet-oedematous and hypertrophic mucosa.

Perforation of the tympanic membrane may also be caused by mastoid related factors. It has been claimed that an occult disease of the mastoid is an important factor in tympanoplasty failure [39]. However, Balyan et al. could not find any significant difference regarding hearing and graft take success in cases with or without mastoidectomy [40]. In our study, there was no significant difference in graft take success in patients who underwent tympanoplasty with or without mastoidectomy. It was also observed that an open or closed aditus in antrotomy and mastoidectomy patients had no significant effect on graft success.

Age is considered as an important factor affecting the surgical success. It is claimed that the surgical success will decrease in the paediatric age group because of the frequent upper respiratory tract infections and the related otitis media [6,8,41]. In contrast, it is also claimed that the child might have developmental disorders caused by the disease and that the Eustachian tube requires a robust eardrum for its functional development [42]. In this study, there was no significant difference between the children and the adults regarding tympanoplasty success. However, at older ages, the success rate decreases, which is thought to be influenced by comorbid diseases and the tissue healing problems.

MERI [13] is a useful tool for the evaluation of the severity of middle ear disease and predicts the surgical success [9,10]. In this study, it can be seen that the anatomic and functional success decreases as MERI scores increase. Moreover, the results showed that MERI scores were related to the type of operation. The highest MERI scores were seen in patients who underwent modified radical mastoidectomy, and the lowest MERI scores were in patients who underwent simple tympanoplasty.

In this study, short-term results were discussed. However, more accurate outcomes can be given with long-term follow-up information.

In conclusion, the aim of the surgical treatment of COM is to provide a well-ventilated, permanently dry ear with closed perforation and improved hearing acuity, and prevent disease recurrences. The treatment comprises eliminating the pathology and reconstructing damaged structures. The factors affecting the success in COM surgeries include age, chronic otitis subgroup, location and size of the perforation, the condition of the middle ear mucosa, and the level of the ossicular disease.

In order not to be disappointed after the operation, the factors affecting the success of surgery should be known, and an appropriate treatment plan should be made.
